# From Surgical Salvage to Blindness Prevention: A Real-World Study of Intraocular Surgery in Monocular Patients

**DOI:** 10.3390/jcm15114041

**Published:** 2026-05-23

**Authors:** Haoxin Guo, Linfei Wei, Gangwei Cheng, Youxin Chen, Rongping Dai, Zhiqiao Zhang, Shunhua Zhang, Xiaoxu Han, Xufeng Zhao, Zaowen Wang, Weihong Yu

**Affiliations:** 1 Department of Ophthalmology, Peking Union Medical College Hospital, Chinese Academy of Medical Sciences, Beijing 100730, China; ghx1054342768@163.com (H.G.); pumcwlf@163.com (L.W.);; 2 Beijing Key Laboratory of Fundus Diseases Intelligent Diagnosis & Drug/Device Development and Translation, Beijing 100730, China; 3 Key Laboratory of Ocular Fundus Diseases, Chinese Academy of Medical Sciences, Beijing 100730, China

**Keywords:** blindness, monocular, intraocular surgery, public health

## Abstract

**Background:** Intraocular surgery on patients with an irreversibly blind fellow eye carries high risks, often causing treatment delays due to patient and surgeon hesitation. Existing data beyond cataracts are scarce. This study aims to evaluate the clinical profiles, prognosis, and economic value of diverse surgeries in this monocular population to guide clinical decision-making and optimize blindness prevention strategies. **Methods:** This retrospective study included 308 patients with a pre-existing blind fellow eye who underwent primary inpatient intraocular surgery under a standardized clinical protocol between June 2021 and June 2025. Baseline demographics, bilateral etiologies, visual outcomes, postoperative complications, and average cost-effectiveness ratios (ACERs) were analyzed. Postoperative outcomes were evaluated for patients with at least 6 months of follow-up. **Results:** The primary surgical indications were cataract (51.3%), proliferative diabetic retinopathy (PDR, 19.5%), glaucoma (15.9%), and rhegmatogenous retinal detachment (RRD, 7.5%). Notably, 49.4% of patients exhibited identical blinding etiologies bilaterally. Among patients completing the 6-month follow-up (*n* = 109), overall mean BCVA significantly improved from 1.36 ± 0.77 to 0.73 ± 0.65 logMAR (*p* < 0.001). The cataract group achieved the greatest visual improvement and the lowest ACER. Despite surgical complexity and higher complication rates, PDR and RRD interventions achieved visual improvement in over 60% of cases. **Conclusions:** Despite high clinical stakes, timely surgery in monocular patients yields substantial visual and economic benefits. The notable disease symmetry highlights a critical window for early intervention, emphasizing the need for public health strategies that prioritize screening progressive bilateral diseases.

## 1. Introduction

Visual impairment and blindness remain profound global public health challenges, severely diminishing patients’ quality of life and imposing substantial socioeconomic burdens [1,2]. Patients with a pre-existing irreversibly blind fellow eye represent a uniquely vulnerable population. The surgical risk–benefit calculus for monocular patients differs fundamentally from that of patients with bilateral functional eyes, as even a single perioperative adverse event can result in bilateral blindness, severely compromising patient independence and escalating long-term care burdens. Consequently, from a public health perspective, optimizing surgical intervention for this high-risk population is a critical imperative for blindness prevention.

In the ongoing global effort against blindness, prompt surgical intervention remains the most critical defense. Existing evidence indicates that timely cataract surgery can restore visual function in monocular patients to levels comparable with binocular cohorts, despite their poorer baseline acuity [3,4,5]. Paradoxically, this imperative for early action is often undermined by immense psychological barriers. Patients undergoing surgery on their only seeing eye endure profound psychological distress [6]. One study revealed that approximately 80% of these patients required over a year to cultivate the psychological readiness to consent to the procedure [7]. Surgeons bear a parallel ethical burden, knowing that even minor operative complications can result in absolute blindness. Consequently, this mutual hesitation severely complicates clinical decision-making, resulting in critical treatment delays that accelerate progression toward the very irreversible blindness they seek to avoid.

Despite the universal consensus on the clinical importance of intraocular surgeries in monocular patients, systematic, large-scale evidence remains remarkably scarce. Current research on this monocular cohort is predominantly confined to cataract surgery [3,4,5], leaving a critical knowledge gap regarding more complex blinding pathologies, such as proliferative diabetic retinopathy (PDR) and rhegmatogenous retinal detachment (RRD). Although these conditions differ pathophysiologically, we analyzed them within a unified framework because operating on the sole functional eye imposes a shared risk-benefit calculus and clinical dilemma across all disease categories. Furthermore, the economic implications and public health value of these high-stakes procedures remain largely unexplored.

To address these gaps, this study presents a large-scale real-world analysis of monocular patients undergoing a diverse spectrum of primary inpatient intraocular surgeries. The objectives of this study are to systematically delineate the demographic and bilateral etiological profiles of this cohort, evaluate the visual prognosis and postoperative complications, and assess the economic value of these interventions. Ultimately, this study will provide crucial evidence-based insights to guide risk-benefit decision-making and optimize public health strategies for blindness prevention.

## 2. Materials and Methods

### 2.1. Study Design and Setting

This was a retrospective, single-center, real-world observational study conducted at the Department of Ophthalmology of Peking Union Medical College Hospital (PUMCH). The study systematically collected and analyzed data from patients with a pre-existing irreversibly blind fellow eye who underwent primary inpatient ophthalmic surgery between June 2021 and June 2025. The study adhered to the tenets of the Declaration of Helsinki and was approved by the Ethics Committee of PUMCH (Approval No. I-25PJ1895). Given the retrospective design and the use of fully de-identified patient data, the requirement for individual informed consent was waived by the committee.

### 2.2. Study Population

Patients were included in this study if they met the following criteria: (1) underwent primary inpatient intraocular surgery during the study period; and (2) had a pre-existing blind fellow eye, defined as a best-corrected visual acuity (BCVA) of counting fingers (CFs) or worse with no potential for visual recovery. Patients with incomplete medical records were excluded from the analysis. For the longitudinal assessment of postoperative outcomes, patients with a follow-up period of less than 6 months were excluded.

### 2.3. Standardized Perioperative Care

A stringent perioperative management protocol was implemented for all patients in this cohort. To facilitate optimal perioperative care and rigorous monitoring, all procedures were performed in an inpatient setting rather than as day surgeries. Preoperatively, patients underwent comprehensive systemic optimization, including strict glycemic and blood pressure control, to ensure surgical safety and expedite timely intervention. Furthermore, the informed consent process was conducted in the presence of legal counsel, requiring the execution of supplementary legally binding documentation to explicitly address the profound prognostic implications and medicolegal risks. All surgical procedures were performed by senior ophthalmic surgeons with extensive expertise in complex ocular surgeries.

### 2.4. Data Collection

Relevant clinical data were systematically extracted from electronic medical records using a standardized collection form. Baseline demographic variables included age, sex, and employment status. The working-age group was defined as 16 to 59 years of age, representing the potential labor force based on China’s official criteria, excluding full-time students. Baseline ophthalmic characteristics comprised bilateral preoperative BCVA, the specific etiology of blindness in the fellow eye, the primary indication for surgery, and the etiological subtypes of major surgical indications. For the analysis of postoperative outcomes, follow-up data comprised postoperative complications and the final BCVA, which was recorded when ocular conditions had stabilized. Additionally, total inpatient hospitalization costs were retrieved to inform the economic evaluation.

### 2.5. Cost-Effectiveness Analysis

Direct medical costs were defined as the total inpatient hospitalization expenses (converted to United States dollars based on the average exchange rate), encompassing fees for surgery, medications, consumables, and accommodation. Clinical effectiveness was measured by the mean reduction in logMAR BCVA from baseline to the final follow-up. To assess the relative economic value, the average cost-effectiveness ratio (ACER), representing the mean cost per 0.1 logMAR improvement [8,9], was calculated for the cataract, PDR, and RRD groups. Patients undergoing glaucoma surgery were excluded from the ACER analysis, as their primary surgical endpoint is intraocular pressure (IOP) preservation rather than visual acuity gain.

### 2.6. Statistical Analysis

All statistical analyses were performed using GraphPad Prism software (Version 10.5.0; GraphPad Software, San Diego, CA, USA). Decimal BCVA measurements were converted to the logarithm of the minimum angle of resolution (logMAR) for statistical analysis. CF, hand movement (HM), light perception (LP), and no light perception (NLP) were assigned logMAR values of 2.0, 2.3, 2.7, and 3.0, respectively [10]. Clinical visual outcomes were categorized into three groups based on the change in logMAR from baseline to the final follow-up: improvement (a reduction of ≥0.3 logMAR), unchanged (a change of <0.3 logMAR), and worsened (an increase of ≥0.3 logMAR) [11]. Continuous variables were presented as the mean ± standard deviation (SD). Categorical variables were expressed as frequencies and percentages. The normality of data distribution was assessed using the Shapiro–Wilk test. The change in BCVA from baseline to the final follow-up was analyzed using a paired *t*-test. Comparisons of continuous variables between two independent groups were performed using an independent samples *t*-test or a Mann–Whitney U test. Categorical variables were compared using the Chi-square test. A two-tailed *p*-value of <0.05 was considered statistically significant.

## 3. Results

### 3.1. Baseline Demographic and Ophthalmic Characteristics

**Figure 1:**
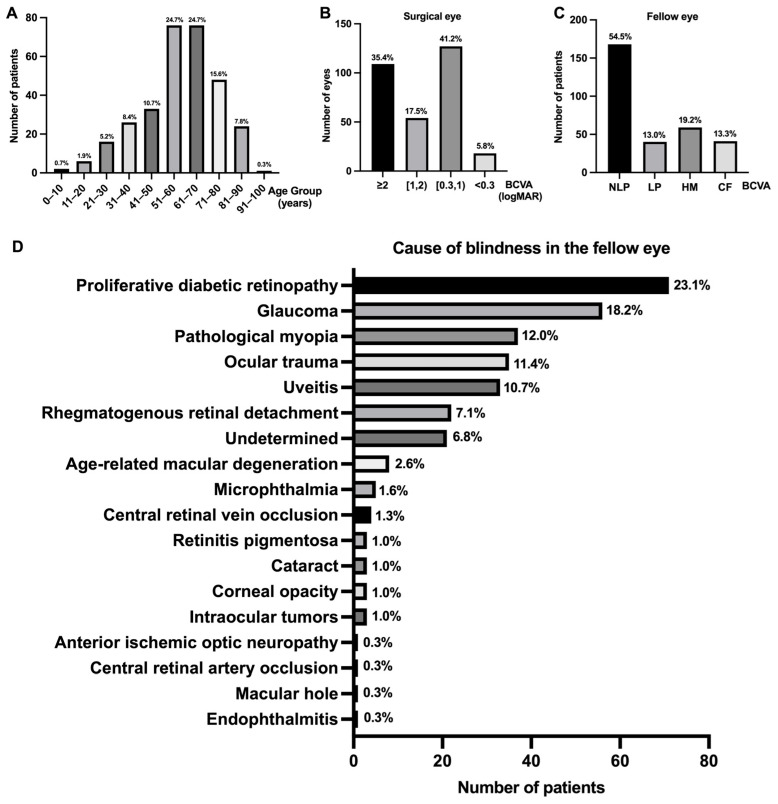
Baseline demographic and ophthalmic characteristics of the study population. (**A**) Age distribution of the monocular patients. (**B**) Preoperative BCVA in the surgical eye. (**C**) Preoperative BCVA in the fellow eye. (**D**) Causes of blindness in the fellow eye.

A total of 308 monocular patients (pre-existing irreversibly blind fellow eye) who underwent primary inpatient intraocular surgery between June 2021 and June 2025 were included in this real-world study. The cohort comprised 164 males (53.2%) and 144 females (46.8%), with a mean age of 58 ± 17 years (range, 10–95 years). The age distribution of the study population is illustrated in Figure 1A. The largest proportions of patients fell within the 51–60- and 61–70-year age groups (24.7% each). Notably, among the 123 working-age individuals, 30 patients (24.4%) reported being out of work at the time of surgery.

The mean preoperative BCVA in the surgical eye was 1.30 ± 0.75 logMAR (range 0.1–2.7 logMAR). Stratified by preoperative BCVA, 109 eyes (35.4%) had a BCVA of ≥2.0 logMAR, 54 eyes (17.5%) were between 1.0 and <2.0 logMAR, 127 eyes (41.2%) were between 0.3 and <1.0 logMAR, and 18 eyes (5.8%) had a BCVA of <0.3 logMAR (Figure 1B). Regarding the fellow eyes, more than half (*n* = 168, 54.5%) presented with NLP. LP, HM, and CF accounted for 40 (13.0%), 59 (19.2%), and 41 (13.3%) eyes, respectively (Figure 1C).

The leading cause of blindness in the fellow eye was PDR (*n* = 71, 23.1%), followed by glaucoma (*n* = 56, 18.2%), pathological myopia (PM) (*n* = 37, 12.0%), ocular trauma (*n* = 35, 11.4%), uveitis (*n* = 33, 10.7%), and RRD (*n* = 22, 7.1%). The causes of blindness in the fellow eye are detailed in Figure 1D. Remarkably, in 152 patients (49.4%), the primary cause of blindness in the fellow eye was the identical pathology responsible for vision loss in the surgical eye.

### 3.2. Surgical Indications and Etiological Characteristics

The primary indication for surgery was cataract (*n* = 158, 51.3%), followed by PDR (*n* = 60, 19.5%), glaucoma (*n* = 49, 15.9%), RRD (*n* = 23, 7.5%), and epiretinal membrane (ERM; *n* = 5, 1.6%). Other less common indications included macular hole (MH), polypoidal choroidal vasculopathy (PCV), and retinal vein occlusion (RVO) (*n* = 2 each). The remaining indications, each accounting for a single case, were cytomegalovirus retinitis (CMVR), ocular trauma, endophthalmitis, familial exudative vitreoretinopathy (FEVR), hypertensive retinopathy, retinal arterial macroaneurysm (RAM), and retinoschisis.

Among the 158 cases of cataract, complicated cataract was the predominant subtype (*n* = 90, 57.0%), primarily secondary to PM (*n* = 46, 51.1%) and uveitis (*n* = 31, 34.4%). Other associated etiologies included microphthalmia (*n* = 8, 8.9%), retinitis pigmentosa (RP), glaucoma (*n* = 2, 2.2% each), and intraocular lymphoma (*n* = 1, 1.1%). Age-related cataract (ARC) accounted for 55 cases (34.8%), while metabolic (diabetic) and toxic (steroid-induced) cataracts accounted for 12 (7.6%) cases and 1 (0.6%) case, respectively.

Within the PDR subgroup (*n* = 60), 41 patients (68.3%) presented with tractional retinal detachment (TRD), and eight (13.3%) had neovascular glaucoma (NVG). Systemically, eight patients (13.3%) had diabetic nephropathy requiring dialysis. Additionally, 16 patients (26.7%) had developed NVG in the fellow eye.

Among the 49 patients with glaucoma, primary angle-closure glaucoma (PACG) was the predominant subtype (*n* = 23, 46.9%), followed by primary open-angle glaucoma (POAG; *n* = 13, 26.5%) and secondary glaucoma (*n* = 13, 26.5%). Secondary glaucoma was attributed to uveitis (*n* = 7, 53.8%), NVG (*n* = 4, 30.8%), and nanophthalmos (*n* = 2, 15.4%).

Furthermore, among the 23 patients with RRD, 18 (78.3%) had concomitant PM. The surgical indications and etiological characteristics of the study population are listed in Table 1.

### 3.3. Visual Outcomes and Postoperative Complications

**Figure 2:**
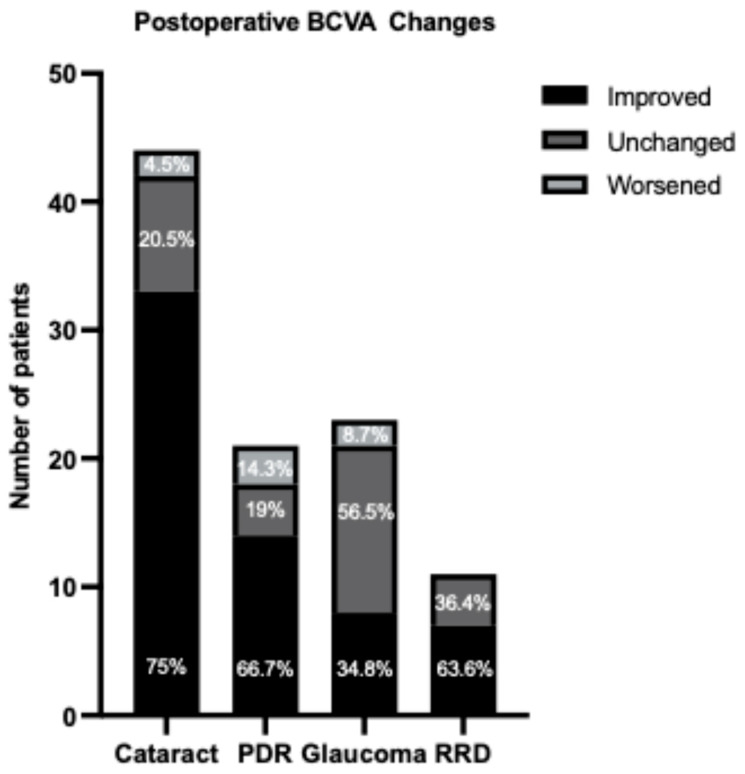
Categorical visual outcomes stratified by primary surgical indication.

Among the 109 patients who completed the 6-month follow-up, the mean BCVA significantly improved from a preoperative value of 1.36 ± 0.77 logMAR to 0.73 ± 0.65 logMAR postoperatively (*p* < 0.001). There were no significant differences in baseline age, sex, or preoperative BCVA between these 109 patients and those lost to follow-up (*p* > 0.05). Stratifying visual acuity outcomes by primary surgical indication across the four major groups revealed the greatest visual gain in the cataract group (*n* = 44) with a mean logMAR reduction of 0.85 ± 0.69. This was followed by the PDR (*n* = 21, 0.71 ± 0.98) and RRD (*n* = 11, 0.62 ± 0.62) groups, whereas the glaucoma group (*n* = 23) exhibited the minimal visual improvement (0.10 ± 0.57). Regarding categorical visual outcomes, visual improvement was observed in 75.0% (*n* = 33) of the cataract group, 66.7% (*n* = 14) of the PDR group, and 63.6% (*n* = 7) of the RRD group. Only 8 patients (34.8%) in the glaucoma group showed visual improvement. The proportions of patients with improved, unchanged, and worsened vision across the four groups are detailed in Figure 2.

Postoperative complications among the followed-up patients are summarized in Table 2. In the cataract group, two patients developed corneal endothelial decompensation. Among patients with PDR, six experienced postoperative complications, comprising NVG (*n* = 3), recurrent vitreous hemorrhage (*n* = 1), recurrent TRD (*n* = 1), and hyphema (*n* = 1). In the RRD cohort, complications included recurrent retinal detachment (*n* = 4) and secondary glaucoma (*n* = 2). Additional isolated complications in other groups included failure of macular hole closure (*n* = 1), pupillary block in a patient with FEVR, and secondary glaucoma necessitating repeat anti-glaucoma surgery in a patient with nanophthalmos.

### 3.4. Economic Evaluation

The mean hospitalization costs varied substantially across the major surgical indications. The average costs were $905 ± 300 for cataract, $2411 ± 560 for PDR, $1286 ± 629 for glaucoma, and $2592 ± 609 for RRD. In the cost-effectiveness analysis, the ACER was lowest for the cataract group at $103 per 0.1 logMAR improvement, followed by the PDR group at $327 and the RRD group at $ 382.

## 4. Discussion

To our knowledge, this study presents one of the largest real-world cohorts comprehensively delineating the clinical profiles of inpatient intraocular surgery in patients with an irreversibly blind fellow eye. Our findings reveal a high prevalence of joblessness (24.4%) among working-age patients, underscoring the profound socioeconomic burden of this condition [12]. Clinically, a notable degree of pathological symmetry between the two eyes was also observed. Despite the inherent high stakes and anatomical complexities, surgical interventions yielded substantial visual recovery across diverse etiologies, with marked variations in cost-effectiveness. These data provide valuable real-world insights for managing this high-risk population.

The formidable clinical and medicolegal stakes inherent in monocular intraocular surgery frequently deter timely intervention. To address these barriers, our institution mandated a rigorous inpatient protocol. This transition from day surgery encompassed stringent systemic health optimization, a fortified medicolegal consent process, and the assignment of senior surgical expertise. While undeniably more demanding, this structured approach appeared beneficial in our experience. It not only minimized perioperative vulnerabilities but also provided the essential clinical and legal scaffolding required to bolster surgeon confidence, thereby facilitating prompt action to rescue the patient’s remaining vision.

A principal finding of this study is the pathological symmetry between the two eyes, with nearly half of the cohort (49.4%) sharing an identical blinding etiology. This concordance is primarily driven by progressive, bilateral diseases, notably PDR and primary glaucoma. Such an observation carries profound public health implications, suggesting that a substantial proportion of this monocular status may be preventable. It underscores a critical, yet frequently missed, window for early intervention in the first-affected eye. Aggressive management of these bilateral pathologies, coupled with enhanced screening and patient education, is imperative to arrest the initial visual decline, thereby pre-empting the formidable scenario of operating on a patient’s sole functioning eye.

While cataract extraction was the predominant surgical indication, our etiological analysis underscores that these were rarely routine procedures. Over half of the cataracts were complicated, primarily secondary to pathological myopia and uveitis, reflecting the complex ocular comorbidities of this cohort [13,14]. Despite this baseline complexity, the cataract group achieved the greatest visual improvement and the most favorable cost-effectiveness. Beyond these objective visual gains, Pomberg et al. demonstrated that monocular patients experienced a near doubling of subjective functional visual improvement (VF-14 scores) postoperatively compared to their binocular counterparts [4]. Given this intersection of functional restoration and economic efficiency, prompt cataract extraction in the sole functional eye represents a viable and beneficial clinical strategy rather than a deferred last resort.

Vitreoretinal surgeries for PDR and RRD represented the most challenging cases in our cohort. Anatomically, the surgical complexity was driven by 68.3% of the PDR subgroup presenting with TRD and 78.3% of the RRD cohort having PM. These structural alterations directly contributed to the high rates of postoperative complications observed. Systemically, 13.3% of PDR patients required hemodialysis, conferring a critical risk of perioperative hemorrhage [15,16]. These compounding factors highlight the need for careful multidisciplinary assessment and rigorous risk stratification before surgery. While the management of PDR and RRD incurred higher ACERs due to complex vitrectomies and prolonged hospital stays, these interventions demonstrated a visual improvement rate exceeding 60% in both cohorts. Nevertheless, a ≥0.3 logMAR improvement may not equate to functional visual recovery. Moreover, the relatively high complication rates in these subgroups—including recurrent retinal detachment in 4 of 11 RRD cases and neovascular glaucoma in 3 of 21 PDR cases—warrant careful consideration of long-term visual stability. Compared to the lifelong socioeconomic burden of bilateral blindness, such as continuous nursing care and loss of patient independence, the upfront inpatient costs may represent a favorable public health investment.

Contrasting our results with existing data from binocular cohorts provides essential context for evaluating the specific prognostic outcomes associated with intraocular surgery in monocular patients. For cataract patients, the UK National Ophthalmology Database (NOD) study reports a mean visual gain of approximately 0.47 logMAR [17]. In contrast, our monocular cataract group demonstrated a more substantial mean improvement of 0.85 logMAR. Despite a high proportion of complicated cataracts (57.0%), this superior visual gain reflects a tendency to defer cataract surgery—given its elective nature—in single-seeing eyes until vision is profoundly impaired. Crucially, this substantial recovery highlights the potential benefits of considering earlier surgical intervention in monocular patients before severe deterioration occurs. In PDR patients, a comparable cohort achieved visual improvement (≥0.3 logMAR gain) in 63.1% of cases following vitrectomy [18]. Our observed rate of 66.7% is highly consistent with this finding, suggesting that the functional success of vitreoretinal surgery in monocular eyes is not inherently compromised by their sole-functioning status. Regarding complications, our RRD cohort exhibited a 36.4% recurrent detachment rate, notably exceeding the 10.5–11.4% range reported in the European Vitreo-Retinal Society (EVRS) study [19]. This elevated recurrence is largely driven by the prolonged surgical hesitation typical of monocular patients. Such delays foster chronic, extensive detachments and a higher incidence of advanced proliferative vitreoretinopathy (PVR)—the primary risk factor for surgical failure [20].

The etiological profile of the glaucoma cohort further exemplifies the pathological landscape of this population. The predominance of PACG aligns with the epidemiological signature of irreversible blindness in the Chinese population [21,22]. Notably, the high proportion of secondary glaucoma highlights the severe immune-mediated and ischemic burdens afflicting these sole functional eyes, predominantly driven by refractory conditions such as uveitis and NVG. Regarding prognosis, the minimal visual improvement observed in the glaucoma cohort reflects the primary therapeutic objective of preserving existing vision through IOP control rather than reversing established optic neuropathy [23]. Consequently, establishing realistic expectations through transparent preoperative counseling is essential to ensure long-term patient adherence and mitigate postoperative psychological distress.

The epidemic prevalence of PM in China presents an escalating public health challenge [24,25]. In our cohort, PM emerged as the primary etiology for complicated cataract (51.1%), the leading comorbidity in RRD cases (78.3%), and the second most frequent cause of absolute blindness in the fellow eye (12.0%). These figures further implicate PM as a critical driver of bilateral vision loss in the Chinese population. Consequently, to pre-empt severe sequelae such as PM-induced RRD and complicated cataract, these findings strongly advocate for proactive myopia control in youth coupled with routine fundus screening in adults with high myopia.

The strengths of this study include its real-world design, a large sample size for this high-risk population, and a comprehensive analysis integrating clinical and economic data. However, several limitations must be acknowledged. The retrospective, single-center nature inevitably introduces selection bias. A total of 64.6% of patients were excluded from the outcome analysis due to insufficient follow-up. Despite comparable baseline characteristics between groups, this attrition may introduce bidirectional bias—driven either by patients seeking alternative care for poor outcomes or deferring visits due to satisfactory recovery. Thus, these prognostic results warrant cautious interpretation. Furthermore, the 6-month follow-up period, while adequate for assessing primary surgical recovery, is insufficient to capture long-term visual outcomes and the incidence of late-onset complications. Lastly, our cost-effectiveness analysis was limited to direct inpatient medical costs and did not incorporate indirect costs or patient-reported outcome measures (PROMs) such as quality of life [26]. Given that monocular patients face unique functional and psychological challenges, the absence of validated instruments such as the NEI VFQ-25 limits the ability to fully capture the clinical significance of visual acuity improvements. Future prospective studies should incorporate PROMs, quality-adjusted life years (QALYs), and longer follow-up periods to provide a more comprehensive assessment.

## 5. Conclusions

In conclusion, while operating on patients with a pre-existing blind eye presents high clinical and medicolegal stakes, our findings demonstrate that timely surgical intervention yields substantial visual recovery and economic value, highlighting the high cost-effectiveness of cataract extraction, with over 60% visual improvement in PDR and RRD cases. The notable disease symmetry (49.4%) between the irreversibly blind eye and the surgical eye highlights a critical, often missed window for early intervention. Preventing patients from reaching this vulnerable monocular state requires public health strategies that prioritize the early screening and aggressive management of progressive bilateral diseases.

## Figures and Tables

**Table 1:** Surgical indications of study population.

Surgical Indication	Eyes, *n* (%)
Cataract (*n* = 158)	158 (51.3)
Complicated cataract (*n* = 90)	90 (57.0)
Pathologic myopia	46 (51.1)
Uveitis	31 (34.4)
Microphthalmia	8 (8.9)
Retinitis pigmentosa	2 (2.2)
Glaucoma	2 (2.2)
Intraocular lymphoma	1 (1.1)
Age-related cataract	55 (34.8)
Metabolic cataract	12 (7.6)
Toxic cataract	1 (0.6)
Proliferative diabetic retinopathy (*n* = 60)	60 (19.5)
With tractional retinal detachment	41 (68.3)
Glaucoma (*n* = 49)	49 (15.9)
Primary angle-closure glaucoma	23 (46.9)
Primary open-angle glaucoma	13 (26.5)
Secondary glaucoma (*n* = 13)	13 (26.5)
Uveitis	7 (53.8)
Neovascular glaucoma	4 (30.8)
Nanophthalmos	2 (15.4)
Rhegmatogenous retinal detachment (*n* = 23)	23 (7.5)
With pathologic myopia	18 (78.3)
Epiretinal membrane	5 (1.6)
Macular hole	2 (0.6)
Polypoidal choroidal vasculopathy	2 (0.6)
Retinal vein occlusion	2 (0.6)
Cytomegalovirus retinitis	1 (0.3)
Ocular Trauma	1 (0.3)
Endophthalmitis	1 (0.3)
Familial exudative vitreoretinopathy	1 (0.3)
Hypertensive retinopathy	1 (0.3)
Retinal arterial macroaneurysm	1 (0.3)
Retinoschisis	1 (0.3)

**Table 2:** Postoperative complications in patients completing follow up.

Surgical indication (*N* = 109)	Postoperative Complications	Eyes, *n*
Cataract (*n* = 44)	Corneal endothelial decompensation	2
Proliferative diabetic retinopathy (*n* = 21)	Neovascular glaucoma	3
	Recurrent tractional retinal detachment	1
	Recurrent vitreous hemorrhage	1
	Hyphema	1
Glaucoma (*n* = 23)	Secondary glaucoma (Nanophthalmos)	1
Rhegmatogenous retinal detachment (*n* = 11)	Recurrent retinal detachment	4
	Secondary glaucoma	2
Macular hole (*n* = 1)	Failure of macular hole closure	1
Familial exudative vitreoretinopathy (*n* = 1)	Pupillary block	1
Epiretinal membrane (*n* = 2)	-	-
Polypoidal choroidal vasculopathy (*n* = 2)	-	-
Retinal vein occlusion (*n* = 2)	-	-
Retinal arterial macroaneurysm (*n* = 1)	-	-
Retinoschisis (*n* = 1)	-	-

## Data Availability

The data of this study are available from the corresponding author upon reasonable request.
